# The role of dobutamine stress magnetic resonance in the clinical management of patients with coronary artery disease

**DOI:** 10.1186/1532-429X-13-S1-O64

**Published:** 2011-02-02

**Authors:** Rolf Gebker, Cosima Jahnke, Robert Manka, Thomas Hucko, Sebastian Kelle, Christoph Klein, Bernhard Schnackenburg, Eckart Fleck, Ingo Paetsch

**Affiliations:** 1 German Heart Institute, Berlin, Germany; 2 University Hospital RWTH, Aachen, Germany; 3 Philips Medical Science, Hamburg, Germany; 4 University Hospital RWTH Aachen, Aachen, Germany

## Background

Dobutamine stress magnetic resonance imaging (DSMR) has consistently high diagnostic and prognostic utility. Its value for clinical decision making still needs to be defined. Hence, the purpose of this study was to assess the role of DSMR regarding clinical management of patients with suspected and known coronary artery disease (CAD) in a routine setting.

## Methods and results

Standard DSMR was performed in 1532 consecutive patients with suspected and known CAD. Patients were stratified according to the results of DSMR: DSMR-positive patients were recommended to undergo invasive coronary angiography and DSMR-negative patients received optimal medical treatment. Of 609 (40%) DSMR-positive patients coronary angiography was performed in 478 (78%) within 90 days. In 409 of these patients significant coronary stenoses (≥50% luminal narrowing) were present (sensitivity 86%). Of 923 (60%) DSMR-negative patients 833 (90%) received optimal medical therapy. During a median follow-up period of 2.1 years 8 DSMR-negative patients (0.96%) sustained a cardiac event.

**Figure 1:**
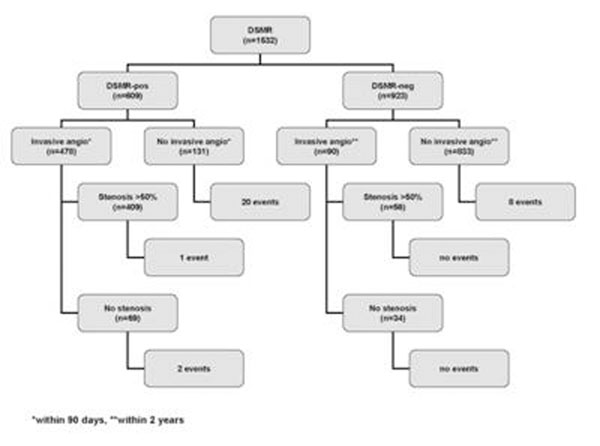


In 131 DSMR-positive patients who did not undergo invasive angiography, 20 patients (15%) suffered cardiac events. In 90 DSMR-negative patients (10%) invasive angiography was performed within 2 years (range 0.01 to 2.0 years) with 56 patients having coronary stenoses ≥50%. Figure 1.

## Conclusion

DSMR can be used as an arbiter in a routine setting for clinical decision making and has high utility for clinical management of patients with suspected and known CAD.

